# Improvement of the qualification system in Korea for foreign nurses eligible to take Korean nursing licensing examination

**DOI:** 10.3352/jeehp.2019.16.26

**Published:** 2019-09-04

**Authors:** Young Whee Lee, Yeon Ok Suh, Kyoung Sook Chae

**Affiliations:** 1 Department of Nursing, Inha University, Incheon, Korea; 2 Department of Nursing, Soonchunhyang University, Cheonan, Korea; The Catholic University of Korea, Korea

**Keywords:** Licensure, Korea, Quality improvement, International nurses

## Abstract

**Purpose:**

We aimed to review and provide a quality improvement for the document utilized by the relevant Korean government body to verify and evaluate foreign university/college graduates’ eligibility for nursing and qualification to take the Korean nursing licensing examination.

**Methods:**

This was a descriptive study. We analyzed the current Korean qualification system for foreign graduates to take the Korean nursing licensing examination and the same system utilized in some other countries. Then, we created a draft of the reviewed qualification standards document based on the 2 prior analyses and their comparisons, and applied a questionnaire in an open hearing with 5 experts to enhance the draft’s quality. Finally, we presented and discussed the final draft.

**Results:**

The reviewed criteria of the qualification standards included confirming whether the foreign graduate’s university has an accreditation provided by its relevant government body, the exclusion of foreign graduates’ provision of several documents previously required, a minimum number of credits (1,000 hours) for their original course, a 3-year minimum enrollment period for their original course, and a mandatory reassessment of the foreign graduates’ university recognition in a 5-year cycle.

**Conclusion:**

We believe that by creating a review draft that addresses the flaws of the current document utilized to determine the qualification for foreign graduates to take the Korean nursing licensing examination, we have simplified it for a better understanding of the application process. We hope that this draft will contribute to a more objective and equitable qualification process for foreign university nurse graduates in Korea.

## Introduction

Recently, due to the development of the healthcare system, the movement of medical personnel among the countries has become active, and the number of healthcare workers flowing into Korea is also increasing. More specifically, Korea has recently experienced an uprising in the contingents of health and medical professionals. Since their practice is directly linked to national health, knowing if these foreign professionals have the required competency to work is of the utmost importance [1]. Starting in 2017, the Medical Law on Nurse Licenses (MLNL) stipulated in Article 7 that only nurse graduates of universities/colleges who have been accredited by the relevant certification body are allowed to take the Korean nursing licensing examination. Thus, it is inferred that domestic students from accredited universities/colleges have the required competency to work because their institutions are certified by the government [2]. Contrastingly, foreign university/college nurse graduates have often completed varied interdisciplinary curriculums, so their competency is not so easily gauged [3].

Therefore, this technical report aimed to review and provide a quality improvement for the criteria within the document utilized by the Korea Health Personnel Licensing Examination Institute (KHPLEI) to evaluate foreign university/college graduates’ eligibility for nursing and qualification to take the licensure exam.

## Methods

### Ethics statement

Expert consultation did not need to receive informed consent because there was no personal information exposure during the consultation process.

### Study design

This descriptive study developed new qualification criteria through the analyses described herein.

### Analysis methods

(1) We thoroughly read and analyzed the whole current Korean qualification system for foreign graduates to take the Korean nursing licensing examination, so as to better understand its criteria and find its flaws/problems.

(2) We analyzed the qualification system for foreign graduates used by other major countries (United States, Japan, Canada, Australia, United Kingdom).

(3) We collected data from 5 experts on the questionnaire that created a draft of the reviewed qualification standards document based on these analyses, its comparisons, and we reviewed the contents through open hearing.

(4) We presented and discussed the final draft of the reviewed qualification standards document—with the newly added criteria—for foreign graduates to take the national licensure exam.

## Results

### Analysis of the Korean nursing competency qualification system for foreign graduates

Regarding qualification criteria, Article 3 of the MLNL states that “the graduate’s university academic system, curriculum, and management levels should be checked, and they must be equal to or higher than the levels found in Korean universities.” However, this is practically difficult to implement because there is no specific regulation regarding criteria that characterizes which university/college system/curriculum/management is “equal to or higher than” that of Korean universities/colleges, so the personal evaluator’s judgment provides this assessment. Therefore, a more specific description of these criteria proves necessary to minimize biased decisions.

Regarding accreditation, domestic nursing universities/colleges must be certified by the Korea Accreditation Board of Nursing Education (KABONE), which is thereby accredited by the Ministry of Education, but foreign universities/colleges usually do not require this type of certification. Thus, it proves necessary to consider whether this is an important qualification criterion that could be used to compare foreign and domestic universities/colleges.

Regarding university/college recognition criteria, Article 5 of the MLNL states that if a recognized university/college has changed its curriculum or is judged to not meet the criteria for recognition any longer, it can be cancelled. However, this is practically difficult to implement because there is no specific regulation regarding the criteria for recognition. Further, there is no expiration date for recognition. Therefore, a more specific description of the license/recognition system criteria proves necessary.

### Analysis of the university/college accreditation and qualification systems for foreign graduates in other countries

Regarding the United States, nationally, there is the Accreditation Commission for Education in Nursing (ACEN) and the Commission on Collegiate Nursing Education (CCNE) [4,5]; furthermore, there are other institutions that perform regional accreditation services. In the United Kingdom, the Nursing and Midwifery Council (NMC) is the only nursing education accreditation body [6,7]. Japan also has an accreditation center, Japan Accreditation Board for Nursing Education (JABNE) [8]. Additionally, relevant institutions in Canada (Canadian Association of Schools of Nursing [CASN]), the United Kingdom, and Australia (Australian Nursing & Midwifery Accreditation Council [ANMAC]), which provide accreditation certificates, also evaluate foreign graduates’ qualification to take on a licensure exam [9-11].

Analysis showed that in Korea, the United States, Canada, and Japan, foreign nurse graduates obtain a license by passing on the national licensure exam; and that in the United Kingdom and Australia they obtain one after completing a specific regulated curriculum that satisfies their requirements to entitle someone as graduated in the nursing area [12,13].

### Analysis of the foreign university/college recognition system

The KHPLEI administers the national licensure exams for 24 healthcare occupations, and it also assesses the recognition of foreign universities/colleges and foreign graduates’ applications and conducts the Korean nursing licensing examination for foreign graduates [14].

According to the KHPLEI, from 2002 to 2018, there were 227 requests for the recognition of foreign universities/colleges, with 33 (14.5%) being disapproved. Furthermore, these universities/colleges presented relevant differences in their systems and the credits required for curriculum completion, which should be utilized while reviewing the recognition criteria. In terms of graduates, the largest number of applications comes from the United States (n=126), followed by Japan (n=64), and Australia (n=14) which refer to the period between 2002 and 2018.

### Expert consultation

Regarding qualification for the Korean nursing licensing examination, all experts agreed that foreign graduates should be allowed to take the exam only if their nursing university/college has been certified by the relevant educational accreditation authority.

Regarding the establishment of the minimum number of credits, one expert responded that it should be established based on the number of credits of the KABONE, while the other 4 responded that it is not appropriate to establish the minimum number of credits. These reasoned that it should not be established based in nursing universities/colleges from European countries—where the allocation of time for course credits varies—and that the difference in academic years would be more appropriate for this type of assessment.

Regarding the need to conduct practice tests prior to the exam, all experts responded it was not necessary. They reasoned it was difficult to accurately assess an individual’s ability by a 1-time practice evaluation, and that it was more appropriate to require an on-site work evaluation over a period of time to obtain a more accurate evaluation.

Regarding recognition re-approval for recognized universities/colleges, 3 experts thought it should be required, and recommended a 5-year cycle as the maximum period for the recognition re-approval system.

Regarding the minimum period for graduation, 4 experts responded that it should be 4 years to comply with the Korean nursing education system that was unified into a 4-year graduation period. Nevertheless, other experts suggested it should be 3 years, based on some universities/colleges in Australia and Europe that have excellent practice-oriented educational systems and a 3-year graduation period.

Regarding foreign graduates’ documents that should be excluded from the list of requirements, all experts responded that the college guidebook should be excluded for both accredited and non-accredited universities/colleges. Furthermore, they responded that course curriculum, lecture syllabus, and college regulations should be excluded for graduates from a foreign accredited university/college.

### Draft of the qualification criteria to assess foreign graduates’ competency in nursing and qualification to take on the Korean nursing licensing examination

Based on the contents of the aforementioned analysis and experts’ opinions, we drafted the qualification standards for foreign graduates’ to take the Korean nursing licensing examination document, as shown in Box 1.

**Box 1.** Draft of the qualification standards for foreign graduates to take the Korean nursing licensing examination

Article 1. (Purpose) The purpose of this document is to establish the standards and procedures for the recognition of foreign universities/colleges in which alumni of the university/college can take the national licensure examination pursuant to Article 5 (1) (3) of the Medical Law.

Article 2. (Scope of application) This standard is applied to students who have graduated from a foreign university/college with a major in nursing, have received a license from the relevant country, and have applied for university/college recognition with the Ministry of Health and Welfare (Hereinafter referred to as the “Applicant”).

Article 3. (Recognition standards) The qualification exam is based on the following criteria, and the detailed review criteria are as shown in Annex 1.

1. The applicant must be a graduate of a nursing university/college with a regular course of 3 years or more.

2. The applicant must be graduated who took courses that correspond to the core subjects of nursing (fundamental, adult, child, maternity, psychiatry, and community nursing, and nursing management).

Article 4. (Recognition evaluation procedures) Evaluation for the recognition of foreign nursing universities/colleges from applicants are conducted by the following procedures.

① Applicants apply for recognition of foreign universities/colleges by attaching the following documents to the application form in Attachment 1. Applicants who have graduated from an accredited nursing university/college must submit documents 1 to 4 for, and applicants for non-accredited university/college must submit documents for each applicant 1 to 6. The term “accredited university” means a university/college that has obtained certification from a government-accredited official body through a series of accreditation procedures; nursing universities/colleges belonging to countries that do not have other accreditation bodies or accreditation bodies are non-accredited nursing universities/colleges.

1. Copy of diploma or certificate of graduation

2. Academic transcripts (including transcripts from nursing colleges before transfer)

3. Copy of license

4. Certificate of immigration

5. Curriculum

6. Lecture syllabus

② The Minister of Health and Welfare decides whether or not to recognize an institution based on the opinion of the Director of Korea Health Personnel Licensing Examination Institute (KHPLEI), and the Minister of Health and Welfare notifies the results to the director of KHPLEI. Finally, KHPLEI notifies the applicant.

③ The yearly review is limited to those who applied until May 31.

Article 5 (Cancellation of recognition) If the recognized university/college is found to not be in conformity with the academic standards due to changes in the academic system, curriculum, and/or management of the university/college, its recognition may be canceled.

A detailing of the aforementioned criteria is shown in Appendix 1.

The following changes were made: the foreign graduates’ provision of certain documents (curriculum, lecture syllabus, college rules, and college guides) have been replaced with the accreditation system, which reflects the certification of an accreditation body and confirms similarities between foreign and domestic universities; the curriculum subjects were changed to include all subjects or contents which related to national nursing license exam; the minimum number of practicing class hours was changed to the standard established by the KABONE (1,000 hours), since there are a limited number of hours allowed to be examined by the competent body in the current standard.

## Discussion

In order to prepare the reviewed qualification standards for foreign graduates to take the Korean nursing licensing examination, the first point analyzed and modified was related to the presence or absence of accreditation from the relevant nursing education authority for the university enrolled to be recognized. The KABONE has been providing accreditations for Korean universities/colleges since 2004, and in the United States (ACEN, CCNE), Canada (CASN), the United Kingdom (NMC), Australia (ANMAC), and Japan (JABNE), the nursing education accreditation system also exists and is functional. Furthermore, as a result of examining the accreditation systems from the United States, Japan, Canada, Australia, and the United Kingdom (countries that have had universities applying for Korean recognition in the last 10 years), it was found that the categories presented in the accreditation documents from these countries did not have the same names of those present in the KABONE document. However, the actual contents were not significantly different from those of the KABONE. Therefore, it seemed appropriate to replace the foreign graduates’ provision of documents—that was required originally required to confirm the similarities between the foreign and domestic universities—for the requirement of analysis of their university accreditation system, as similar characteristics were found between foreign and domestic accredited universities, and this modification greatly simplifies the whole process. Furthermore, if this is modified, it should be verified that the applicant’s admission date is in consonance with the accreditation period (that it has not expired) of the relevant university/college, and this accreditation must have been provided by a government-recognized institution.

The second point was related to the minimum number of practice hours. When comparing the course credits of foreign nursing universities/colleges by country, we found that each nursing university/college had different graduation requirements: some were defined by credits, and others were defined by unit. Regarding the US and Japan universities, there is a total of 120–130 credits for each course, a number similar to that of domestic nursing universities/colleges [2]. Additionally, there were varied types of nursing education systems; in the United Kingdom and Australia, there was some conflicting information as to whether graduates take their basic courses related to their major at the A-level (considered the university basic course); hence, these countries determine that a required minimum number of credits is not an appropriate measure to evaluate this level of education. However, since nursing science is practice-oriented, the results had demonstrated that it was still necessary to evaluate graduates’ hours of practice/credits. Thus, the minimum practice time of 1,000 hours (a number determined by the KABONE) was included in the qualification standards. Furthermore, it is suggested that the KABONE should carry out a research to analyze the minimum curriculum contents/subjects to be utilized in the qualification standards document.

The third point was related to the minimum enrollment period for graduation. Currently, most Korean nursing universities/colleges are unified under the 4-year graduation system, and fewer others are slowly adhering to it. However, if 4-year graduation was to be required, universities/colleges from certain countries (like the United Kingdom and Australia), where 3-year graduation is the standard, would have been automatically excluded, despite having well-established practice-oriented curriculums; as for the 3-year program US graduates, it was discussed that they could request for a 1-year study/work process within Korea to be considered competent, but these solutions incur other problems, such as the establishment of a domestic institution(s) capable of providing these experiences. Therefore, it was decided that the appropriate criteria for the minimum enrollment period would be 3-year graduation, so as to not exclude known competent graduates from diversified regions/universities/colleges.

The fourth point was related to the university recognition period. Currently, once a university is recognized, the title remains definitely, and there are no requirements or a period for reevaluation. However, since various aspects of the university can change over time, there is a necessity for reassessment over cycled periods of time. In response, the consulted experts stated that the recognition reassessment should be set to 5 years based on the KABONE accreditation reassessment cycle, which is held every 5 years. Additionally, it will be necessary to verify that the recognition is activated at the time of application.

In conclusion, we examined the flaws/problems present in the current qualification standards for foreign graduates to take the Korean nursing licensing examination, and drawn up a revised draft that addressed these flaws. We believe it has simplified the document and will most likely provide a better understanding of the process for foreign graduates. We hope that this draft will contribute to a more objective and equitable application procedure between foreign university graduates and the KHPLEI.

## Supplementary materials

Supplement 1. Audio recording of the abstract.

## Appendix 1. Detailing of the criteria used in the qualification standards for foreign graduates to take the Korean nursing licensing examination (draft)

**Table:**

Category	Criteria	Qualification standards
Regarding the applicant’s foreign country systems	Licensing system	The applicant must have a valid license (without any information that limits its validity).
		The applicant’s acquired license has the same effects as the domestic one. (Any foreigner who is licensed in her/his country may be employed or practice in the relevant industry within Korea)
	Accreditation system	The applicant’s respective nursing university/college must have been certified by an accreditation authority from its respective government.
Regarding the academic characteristics of the applicant’s university	Academic system: curriculum	The applicant’s curriculum must comply with all courses present in the national licensure exam.
	Academic system: practice hours	The applicant must have completed the clinical practice hours correspondent to the criteria presented by the Korea Accreditation Board of Nursing Education (1,000 hours).
	Admission and transfer procedures	The applicant must comply with the number of foreigners and the admission/transfer procedures that are stipulated within the desired university/college regulation.
		The applicant’s course must either be recognized at the time of transfer or be approved after an evaluation of compliance with domestic regulations through reasonable procedures.
		The foreign graduate applying for admission or transfer should verify his/her language ability regarding the country concerned or go through the domestic language school.
	Credit recognition	The applicant’s university/college regulations must stipulate a required number of credits, and the graduate confirms he has complied with the required attendance days for each semester.
	Extraordinary special course	The applicant’s curriculum must not have an extraordinary special course for foreigners, and it should be equivalent to a national curriculum.
Regarding the applicant	Degree acquisition	The applicant must have graduated from the university and earned a suitable degree/major.
	Academic period	The applicant’s required academic semester and year must comply with the Korean academic semester and year.
		Considering the period of stay and the period of registration, the curriculum that the person has completed is appropriate.
	Current status of completion of course subjects	The applicant must have completed all courses corresponding to the compulsory subjects of nursing (fundamental, adult, child, maternity, psychiatry, and community nursing, and nursing management).
		The applicant must have studied and completed course subjects that are consistent and correlated to the subjects present at Korean nursing universities/colleges.
	Study abroad procedures	The applicants’ admission/transfer procedures must have been legitimate, including the student visa obtaining.
	Earned license	The applicants’ must have earned a nurse’s license.
